# Phenolic phytochemistry, *in vitro*, *in silico*, *in vivo*, and mechanistic anti-inflammatory and antioxidant evaluations of *Habenaria digitata*

**DOI:** 10.3389/fphar.2024.1346526

**Published:** 2024-02-29

**Authors:** Hassan Hussain Almasoudi, Muhammad Saeed Jan, Mohammed H. Nahari, Abdulfattah Yahya M. Alhazmi, Abdulkarim S. Binshaya, Osama Abdulaziz, Mater H. Mahnashi, Muhammad Ibrar, Rehman Zafar, Abdul Sadiq

**Affiliations:** ^1^ Department of Clinical Laboratory Sciences, College of Applied Medical Sciences, Najran University, Najran, Saudi Arabia; ^2^ Department of Pharmacy, Bacha Khan University, Charsadda, Pakistan; ^3^ Pharmaceutical Practices Department, College of Pharmacy, Umm Al-Qura University, Makkah, Saudi Arabia; ^4^ Department of Medical Laboratory Sciecnes, College of Applied Medical sciences, Prince Sattam bin Abdulaziz University, Alkharj, Saudi Arabia; ^5^ Clinical Laboratory Sciences Department, College of Applied Medical Sciences, Taif University, Taif, Saudi Arabia; ^6^ Department of Pharmaceutical Chemistry, Pharmacy School, Najran University, Najran, Saudi Arabia; ^7^ Department of Pharmaceutical Chemistry, Faculty of Pharmaceutical Sciences, Riphah International University, Islamabad, Pakistan; ^8^ Department of Pharmacy, Faculty of Biological Sciences, University of Malakand, Chakdara, Khyber Pakhtunkhwa, Pakistan

**Keywords:** phenolic, mechanism, antioxidant, anti-inflammatory, *Habenaria digitata*, molecular docking

## Abstract

Excessive and imbalance of free radicals within the body lead to inflammation. The objective of the current research work was to explore the anti-inflammatory and antioxidant potential of the isolated compounds from *Habenaria digitata.* In this study, the isolated phenolic compounds were investigated for *in vitro* and *in vivo* anti-inflammatory potential along with the antioxidant enzyme. The anti-inflammatory and antioxidant potential of the phenolic compounds was assayed via various enzymes like COX-1/2, 5-LOX and ABTS, DPPH, and H_2_O_2_ free radical enzyme inhibitory assay. These compounds were also explored for their *in vivo* antioxidant activity like examining SOD, CAT, GSH-Px, and MDA levels in the brain, heart, and liver. The anti-inflammatory potential was evaluated using the carrageenan-induced pleurisy model in mice. On the basis of initial screening of isolated compounds, the most potent compound was further evaluated for the anti-inflammatory mechanism. Furthermore, the molecular docking study was also performed for the potent compound. The phenolic compounds were isolated and identified by GC-MS/NMR analysis by comparing its spectra to the library spectra. The isolated phenolic compounds from *H. digitata* were 5-methylpyrimidine-24,4-diol (1), 3,5-dihydroxy-6-methyl-2,3-dihydropyran-4-one (2), 2-isopropyl-5-methylphenol (3), 3-methoxy-4-vinylphenol (**4**), and 2,6-dimethoxy-4-vinylphenol (5). In *in vitro* antioxidant assay, the most potent compound was compound **1** having IC_50_ values of 0.98, 0.90, and 5 μg/mL against ABTS, DPPH, and H_2_O_2_, respectively. Similarly, against COX1/2 and 5-LOX ,compound **1** was again the potent compound with IC_50_ values of 42.76, 10.70, and 7.40 μg/mL. Based on the *in vitro* results, compound **1** was further evaluated for *in vivo* antioxidant and anti-inflammatory potential. Findings of the study suggest that *H. digitata* contains active compounds with potential anti-inflammatory and antioxidant effects. These compounds could be screened as drug candidates for pharmaceutical research, targeting conditions associated with oxidative stress and inflammatory conditions in medicinal chemistry and support their ethnomedicinal use for inflammation and oxidative stress.

## 1 Introduction

Antioxidants are a type of defensive measure that protects a physiological system from the damaging impacts of oxidative reactions caused by reactive oxygen species (ROS) (Hasanuzzaman et al., 2020). ROS are not only formed spontaneously in cells during respiration and stress, but they have also been linked to bacterial toxins, radiations, viral toxins, alcoholism, smoking, and emotional and psychological stress (Sadiq et al., 2015). Excess synthesis of ROS and/or a deficiency in antioxidants were linked to the progression and severity of illnesses, i.e., Alzheimer’s disease, atherosclerosis, cancer, arthritis, diabetes, neurological illness, and other pathological conditions (Tirichen et al., 2021). Antioxidants were established to protect cells from oxidative damage produced due to ROS through serving as oxygen scavengers and reacting with free radicals, catalytic metals, and chelating agents. Enzymatic and non-enzymatic antioxidants are found in physiological systems (Adwas et al., 2019). Glutathione, superoxide dismutase, and catalase are enzymatic antioxidants that are responsible for the neutralization of various forms of free radicals, whereas polyphenols, carotenoids, selenium, vitamin C, and vitamin E are non-enzymatic free radical scavengers (Zafar et al., 2021). Antioxidants appear to play a key role in protection from pulmonary disease, heart disease, neurological disorders, cancer, and DNA deterioration, according to mounting research. The medicinal significance of herbs as antioxidants in decreasing oxidative cell damage has now sparked increasing attention (Jabeen et al., 2018). Natural herbs and spices that are high in phenolic contents such as flavonoids were shown to have anti-aging, anti-carcinogenic, anti-allergenic, anti-inflammatory, and anti-viral effects that might be associated with the antioxidant characteristics of phenolic compounds (Shah et al., 2014a; Jan et al., 2020).

Several parameters, like food, depression, and environmental variables, have recently enhanced the prevalence of numerous inflammatory disorders (Shah et al., 2014a). Inflammation is a complicated phenomenon that is commonly linked to pain, which encompasses processes like protein denaturation, increased vascular permeability, and membrane modification (Jan et al., 2020). Histamines, bradykinins, and prostaglandins are released whenever tissue cells are damaged. Chemotaxis is a phenomenon in which various compounds function as chemical signals that influence most of the body’s natural defense systems (Alam et al., 2020). Chronic inflammation is defined by a gradual alteration in the kinds of cells available at the inflammation site, as well as simultaneously damaging and repairing of the tissue as a result of the processes involved in systemic inflammation (Alshehri et al., 2024).

Amongst the biologically active components found in *Habenaria digitata*, components with antioxidant activities, including polyphenols, have attained significant interest from academic researchers because of their involvement in the mitigation of disorders related to oxidative stress (Alshehri et al., 2022). The principal bioactive constituents of Orchidaceae are polyphenols, which play a key role in its pharmacological action. The ability of phenolic compounds to maintain cell membrane integrity via free radical scavenging and decreasing lipid peroxidation is among its significant advantages (Mahnashi et al., 2021).

Inflammation and free radicals have a direct connection and can expand collectively. During inflammatory processes, excessive free radicals are produced within the body, which is beyond the limits of the defense system to control it. These excessive free radicals further increase and complicate inflammation. Therefore, to control inflammation within the body, it is also necessary to combat the free radicals to avoid expansion in inflammatory process.

In ethnomedicine, the family Orchidaceae is renowned and is used in traditional medicines in a lot of areas. Since thousands of years, orchids have been used in conventional medication for the treatment of various medical problems such as gastrointestinal dysfunction, jaundice, acidity, arthritis, piles, syphilis, sexually transmitted diseases, boils, wounds, cholera, blood dysentery, tuberculosis, earache, tumor, malaria, hepatitis, eczema, vermifuges, diarrhea, inflammations, and as antioxidants (Kong et al., 2003; Hossain, 2011; Ramos et al., 2012). The much more prominent ethnopharmacological applications of Orchidaceae are anti-inflammatory, antioxidant, and analgesic properties (Anilkumar, 2010; Apu et al., 2012; Barragán-Zarate et al., 2020). The biochemical components of Orchidaceae species have been identified to exhibit significant pharmacological effects (Wu et al., 2019). Species of the orchid family, such as *Microstylis wallichii* and *Vanda roxburghii*, were found to have significant analgesic, antioxidant, and anti-inflammatory properties due to its highest ethnopharmacological heritage (Reddy et al., 2007; Begum et al., 2018). Despite being a renowned Orchidaceae species, no research on the isolation of *H. digitata* components has been published. As a result, the present investigation aims to confirm the particular mechanisms of phenolic compounds behind the anti-inflammatory and antioxidant effects extracted from *H. digitata*.

## 2 Materials and methods

### 2.1 Chemical reagents and drugs

The study’s solvents (analytical grades), chemical reagents, and drugs all were bought from Sigma-Aldrich’s local distributor. Human recombinant 5-LOX (catalog number 437996), cyclooxygenase (COX-2) source human recombinant (catalog number C0858), COX-1, arachidonic acid (CAT No 150384), and linoleic acid (CAS No. 60-33-3). *N*, *N*,*N*,*N*-tetramethyl-p-phenylenediamine dihydrochloride (TMPD) (CAS 637-01-4), indicators, co-factor substances, hematin (CAS No: 15489-90-4), glutathione (CAS 70-18-8), arachidonic acid, bradykinin, DPPH, ABTS, H_2_O_2_, leukotriene, and histamines all were ordered from Sigma-Aldrich’s local distributor.

### 2.2 Plant collection and preparation of extracts

*H. digitata* was collected in the month of April from different areas of Dir (L) KPK, Pakistan, recognized by Prof. Muhammad Nisar, University of Malakand. The plant specimen has been reserved and assigned the voucher code H.UOM.BG.180 at the herbarium. The upper segments of the gathered plant (16 kg) were cleaned and washed (sterile water) and shade-dried for 3 weeks. The dried sections were first sliced into tiny chunks and then crushed into a loutish powder with a blender (7.5 kg). For 3 weeks, the pulverized product was macerated in 80 percent methanol (26 L). Thereafter, it was filtered through a muslin cloth succeeded by a Whatman filter paper. The filtrate was concentrated at 40°C using a rotary evaporator. An amount of 650 g of a dark greenish solid methanolic extract (Hd.Cr) was produced (Sadiq et al., 2020).

### 2.3 Fractionation

Hd.Cr was carefully transferred to a separating funnel and diluted with 500 mL each of water and *n-hexane*. To thoroughly mix the ingredients, the separating funnel was quickly agitated and thereafter placed in a holder at the appropriate level to generate two discrete levels, the n-hexane and aqueous layers. The n-hexane portion was separated. The method was performed twice again using n-hexane (500 mL) each time. All three organic phases were mixed and condensed using a rotary evaporator under reduced pressure, set at 40°C. The acquired Hd.Hex’s weight was 27.5 g. Upon changing the polarity of the solvents, the exact technique was performed with different solvents. Ethyl acetate, chloroform, and n-butanol were the subsequent solvent fractions recovered, having weights of 33, 47, and 89 g, respectively. Approximately 140 g weight of the aqueous layer was finally concentrated (Shah et al., 2014b).

### 2.4 Isolation of phenolic compounds

Column chromatography was used to isolate probable chemicals from ethyl acetate fractions depending on earlier findings. We initiated the chromatographic procedure with only n-hexane at first (100 percent) by increasing polarity elution systems. When sufficient time has passed, we gently increased the polarity by adding 2% ethyl acetate every time (i.e., 98: 2 96: 4 94: 6 92: 8 90: 10). Each time, the TLC was visualized, and the polarity was adjusted correspondingly. Then, based on the number of fractions, we chose columns of large size that were packed with silica slurry containing the desired fractions. Elution began with nonpolar n-hexane and progressed to increased polarity with the introduction of ethyl acetate. Partially pure fractions (about 80%) were obtained, processed on TLC plates to produce combined co-elution fractions depending on R*f* values, and then purified further using silica-packed pencil columns. For the purification of target components, the columns were eluted with chloroform and n-hexane solvent systems once again. The phytochemicals (**1–5**) were recovered from the bioactive fraction of ethyl acetate. The purified quantities of compounds **1–5** were 251, 277, 124, 147, and 271 mg, respectively (Farooq et al., 2019).

### 2.5 *In vitro* antioxidant assay

#### 2.5.1 Antioxidant studies using DPPH assay

The DPPH (anti-radicals) investigation was carried out according to our earlier reported protocols. Initially, a methanolic DPPH solution was prepared with a concentration of 0.1%. Subsequently, 100 µL of this solution was mixed with an equal volume of the test samples (100 µL) in 96-well plates, following a 30-min incubation period at 25°C ± 3 °C in the dark. Solutions with concentrations ranging from 1,000 to 62.5 g mL^−1^ were prepared. Five concentrations of 1,000, 500, 250, 125, and 62.5 *µ*M of the Trolox methanolic solution (100 *µ*M) were formed. A color reduction in DPPH was observed after incubation; a microplate reader was used, and absorbance was measured at 540 nm (Mahnashi et al., 2022a; Huneif et al., 2022). The percentage of scavenging was determined by the formula:

$$\text{Scavenging effect }\%=\frac{\text{control}\;\text{absorbance}-\text{sample}\;\text{absorbance}}{\text{control}\;\text{absorbance}}\times 100.$$

#### 2.5.2 Antioxidant activity via ABTS assay

The ABTS anti-radicals’ analysis was carried out according to Shah et al. (2015). The ABTS solution was prepared by dissolving ABTS salt (192 mg) in distilled H_2_O, transferred to a flask (50 mL), and volume was adjusted. Thereafter, 1 mL of the preceding solution was added to 17 *µ*L of K₂S₂O₈ (140 mM) and placed in the dark for 24 h s. The experiment’s ultimate ABTS dilution was achieved by mixing 1 mL of the reaction mixture with 50 mL of methanol. The ABTS solution (190 *µ*L) was mixed with the sample solution (10 *µ*L) and added to 96-well plates, which were then incubated at room temperature in dark for 2 h. Following incubation, the measurement of ABTS color intensity was conducted using a microplate reader at 734 nm. Positive control solutions were prepared at values of 1,000–62.5 *µ*M. The percentage of ABTS scavenging was determined by using the following equation:

% ABTS scavenging=Control−Sample/control×100.

#### 2.5.3 H_2_O_2_ free radical scavenging activity

The antioxidant potential of isolated compounds was determined using this approach. In a phosphate buffer solution (50 mM, pH 7.40), a solution of H_2_O_2_ (2 mM) was produced. The crude extract (0.10 mL) was measured in a test tube and diluted to 0.40 mL using phosphate buffer. Thereafter, H_2_O_2_ (0.60 mL) was added and thoroughly mixed. A total of 230 nm was used to measure the absorbance of the sample and the standard (Sadiq et al., 2018). For all the crude samples, the exact process was followed. According to the procedure, the inhibition percentage and IC_50_ values were computed.

### 2.6 *In vitro* anti-inflammatory assay

#### 2.6.1 Cyclooxygenase (COX-1/2) assay

The COX-2 inhibitory effect was determined using the procedure previously described (Sadiq et al., 2021). The COX-2 enzyme solution with 300 U/mL concentrations was formed. The enzyme solution (10 µL) was maintained on ice up to 10 min for activation. Moreover, this solution was added to a 50 µL co-factor solution containing 1 mM hematin, 0.9 mM glutathione, and 0.24 mM N,N,N,N-tetramethyl-p-phenylenediamine dihydrochloride (TMPD) in 0.1 M Tris HCl buffer at pH 8.0. Following that, 20 µL of test samples with different concentrations (31.25–1,000 μg/mL) and the enzyme solution (60 µL) were maintained at 25°C for 5 min. Likewise, 30 mM of arachidonic acid (20 µL) was mixed for initiating the reaction and incubated for 4–5 min. Following incubation, absorbance of the samples was determined using a UV-visible spectrophotometer at 570 nm. The percentage inhibition of the COX-2 enzyme was determined through the absorbance value per unit time. Enzyme inhibition against various concentrations of investigated samples was used to calculate the IC_50_ values. Celecoxib was selected as the standard medication in the trial.

#### 2.6.2 5-Lipoxygenase (5-LOX) assay

The 5-LOX inhibitory experiments on *H. digitata* samples were carried out as per published protocol (Pervaiz et al., 2022). Several concentrations of plant samples were made, with concentrations ranging from 31.25 to 1,000 μg/mL. Thereafter, 5-LOX was primed with 10,000 U/mL solutions. Linoleic acid (80 mM) was used as the substrate. Likewise, 50 mM phosphate buffer with a pH of 6.3 was primed. Plant samples of varied quantities were dissolved in 250 µL of phosphate buffer, and then, 250 µL of the lipoxygenase enzyme solution was added and incubated for 5 min at room temperature. A volume of 1,000 µL of the substrate solution with a concentration of 0.6 mM was added to the enzyme solution and shaken. The absorbance at 234 nm was observed. All of the tests were conducted three times. In this test, zileuton served as a positive control. The percentage inhibition was determined using the following equation:

$$\text{Percentage Inhibition}=\frac{\text{Control}\;\text{Abs}.\;-\text{Sample}\;\text{Abs}.\;}{\text{Control}\;\text{Abs}.\;}\times 100.$$

The IC_50_ values were derived by plotting the inhibitions against the concentrations of the tested sample solution.

### 2.7 Experimental animals

Swiss albino mice of either sex (25–30 gm) used in the pharmacological studies were obtained from the National Institute of Health (NIH), a research laboratory in Islamabad, Pakistan. According to the ethical committee’s approval, all mice were placed in an animal house with a standard diet and a dark/light cycle. The experimental animals were utilized with the formal agreement of the ethical committee via a letter (no: UOS-02/2022) at the Department of Pharmacy, University of Swabi, as well as the Animal Bye-laws of 2008. The mice were euthanized by conventional techniques after the pharmacological studies (Leary et al., 2013).

### 2.8 Acute toxicity study

The experimental albino mice were used in the acute toxicity test that were separated into control and test groups (*n= 5*). The *H. digitata* extract was administered orally at various doses (ranging from 25 to 500 mg/kg body weight) prepared in a Tween-80 solvent. Animals were monitored for aberrant behavior and minor allergy reactions for up to 72 h after receiving the doses (Rahim et al., 2017).

### 2.9 *In vivo* antioxidant assay of the potent compounds

#### 2.9.1 *In vivo* antioxidant potential

Twenty male albino mice were distributed into four groups, each with five mice. The control group (group 1) received 0.4 mL of distilled water. The compound was given in doses of 2.5, 5, and 10 mg/kg to groups 2, 3, and 4, respectively. Throughout the trial, the mice were given a daily dose for 3 weeks and were evaluated for alterations, other symptoms of toxicity, and death on a daily basis. Blood obtained by a direct heart puncture was utilized to assess the compound’s *in vivo* antioxidant activity 24 hours following the last dose (Mota et al., 2023).

#### 2.9.2 Analytical methods

##### 2.9.2.1 Serum preparation

Following moderate chloroform anesthesia of the mice, blood was taken through cardiac puncture with a 5-mL syringe (21 G needle) for serum preparation. Yesufu et al. established a standard procedure for preparing the serum. In a nutshell, the process involves the following steps. Blood was left to coagulate before being centrifuged for 15 min at 2,500 rpm to extract the serum.

##### 2.9.2.2 Lipid peroxidation (LPO) determination in the serum

The production of thiobarbituric acid reactive substance (TBARS) and malondialdehyde (MDA) in the serum was determined using a modified method (Ghani et al., 2022). A measure of 1 mL of trichloroacetic acid (14%) and 1 mL of thiobarbituric acid (0.6%) were added to the serum (50 μL) to deproteinize it. To finalize the reaction, the mixture was placed in a water bath, heated for 30 min, and then chilled on ice for 5 min. Absorbance of TBARS was determined with a UV spectrophotometer at 535 nm after centrifugation at 2000 *g* for 10 min. The TBARS concentration was determined using the malondialdehyde molar extinction coefficient (1.56 × 10^5^ mol/L/cm) using the following formula:

A=∑CL,

where A represents absorbance, Σ represents the molar coefficient, C represents the concentration, and L represents the path length. The results were presented as nmol/mg of protein.

##### 2.9.2.3 Superoxide dismutase (SOD) estimation

The activities of SODs were measured using the Sun et al. technique (Uddin et al., 2015). In this procedure, a superoxide flux was produced using the xanthine–xanthine oxidase system, and nitroblue tetrazolium (NBT) was utilized as a superoxide generation indicator. The degree of inhibition of the reaction unit of the enzyme that provided 50% inhibition of NBT reduction was then used to determine SOD activity. The results are given in units of U/mL.

##### 2.9.2.4 Estimation of catalase activity

The altered approach reported by Atawodi was used to determine the catalase activity in the serum (Landahl, 1953). In a nutshell, the process involves the following steps: 10 μL of the serum was added to a test tube having 2.80 mL of potassium phosphate buffer (pH 7.0) with a concentration of 50 mM. The reaction was started by adding 0.1 mL of fresh 30 mM H_2_O_2_, and the decomposition rate of H_2_O_2_ was measured on a spectrophotometer at 240 nm for 5 min. Catalase activity was determined using a molar extinction coefficient of 0.041 mM^−1 ^cm^−1^.

##### 2.9.2.5 Determination of GSH

The brain, liver, and heart homogenates were prepared in the cold saline via a homogenizer, and the unsolvable extract was discarded by centrifugation at 10,000 rpm for 15 min at temperature 4°C. After that, the supernatant was extracted immediately for GSH-Px assay (Li et al., 2021).

### 2.10 *In vivo* anti-inflammatory assay

#### 2.10.1 Carrageenan-induced *in vivo* inflammation

Mice of both sexes (25–30 g) were used for *in vivo* anti-inflammatory activity of test samples on the carrageenan-induced inflammation model (Munir et al., 2020). Mice were distributed into five groups, with eight animals in each group, divided at random. Group-I served as the negative control and received DMSO at a dose of 10 mL/kg body weight. Group-II served as a positive control, which received aspirin at a dose of 100 mg/kg body weight. *H. digitata* samples were administered at a dose of 25, 50, and 75 mg/kg to groups III, IV, and V, respectively. Each mouse received 1 percent (w/v) saline solution (0.05 mL) of carrageenan in the sub-planter region after a 30-min interval. The paw edema volume was determined using a plethysmometer (LE 7500-plan lab S.L) at 1–5 h intervals after carrageenan was given. The paw edema of the investigated plant samples and the standard medication were noted at various intervals of time and compared to the negative control group (Jan et al., 2020). The percentages of inhibition of inflammation were calculated using the formula given below:

$$\%\text{ inhibition}=EC-\frac{ET}{EC}\times 100,$$

where “EC” is the average edema of control and “ET” is the edema of the tested group.

#### 2.10.2 Mechanism of inflammation of the potent compounds

Employing prostaglandin E2, bradykinin, leukotriene, and histamine induced paw edema assays; the potential anti-inflammatory mechanism of the isolated compound was investigated. BALB/c mice (25–30 g) of both sexes were injected intraperitoneally with (i.p.) 10% DMSO or Bradykinin inhibitor (HOE 140), 1 mg/kg lipoxygenase inhibitor (montelukast) or chlorpheniramine maleate, 25 mg/kg anti-histamine, 100 mg/kg of the tested compound (100 mg/kg), or 50 mg/kg of the cyclooxygenase inhibitor (celecoxib). Sub-planter injections of prostaglandin E_2_ (0.01 mg/mL) or 10 mg/mL leukotriene or bradykinin (20 mg/mL) or 0.1 mL histamine (1 mg/mL) produced paw edema after 1 h. Each mouse’s paw volume was assessed immediately before and after sub-planter injection of various irritants (inflammatory agents) at 1–5 h.

### 2.11 *In silico* studies

In the current study, we conducted *in silico* docking investigations using PyRx, connected to AutoDock Vina 1.2.2, to analyze how the synthesized compounds bind with the target site. The molecular structures of the synthesized compounds and the reference standard were sketched using ChemDraw 20.0, the most recent software version, and saved in Molfile format (Waseem et al., 2022). After incorporating polar hydrogen atoms, structures were modified, and they were subsequently filed in PDB form using the Discovery Studio Visualizer. Concurrently, 3D configurations of the designated proteins were obtained from the RCSB Protein Data Bank (http://www.rcsb.org). The COX proteins, encompassing cyclooxygenase I, II, and 5-lipoxygenase, were retrieved with identifiers 4O1Z, 5F1A, and 3V92, respectively. The obtained structures underwent optimization using BIOVIA Discovery Studio Visualizer, involved the addition of polar hydrogen atoms and removal of co-crystallized ligands. Subsequently, the optimized structures were saved in PDB format. To enhance their accuracy, energy minimization was performed on both the macromolecule structures and synthesized ligands using the CHARMM force field, refining any unfavorable crystallographic observations.

Following these steps, the synthesized compounds and targeted proteins were imported into PyRx, where they were modified into ligand and macromolecule formats. A grid box was positioned at coordinates X: 10.132, Y: 66.509, and Z: 32.9381, with dimensions (in Ångströms) of X: 77.0983, Y: 106.8761, and Z: 105.4147. The ligands, when they docked into the receptors’ active sites, were recognized by specifying the co-crystallized ligand pockets. Diverse factors were employed to know optimal interactions, type, and distance of bonds and amino acid residues. These findings were visualized using Discovery Studio Visualizer and PyMOL 1.8, utilizing various colors to represent different aspects of the interactions.

### 2.12 Determination of IC_50_ values

The concentrations of test samples that caused the inhibition of substrate hydrolysis by 50% (IC_50_) were estimated using the Microsoft Excel application.

### 2.13 Statistical analysis

All experiments were carried out in triplicate, and findings were represented as mean SEM. GraphPad Prism software (United States) was used to perform a one-way ANOVA, followed by a Dunnett’s multiple comparison test to compare the positive controls with the test group (Huneif et al., 2022). *p* values of less than 0.05 were deemed statistically significant. All *in vitro* assay values are given as mean SEM with n = 3. The *p*-values were compared to the standard medication, for example, * = *p* < 0.05, ** = *p* < 0.01, and *** = *p* < 0.001.

## 3 Results

### 3.1 Structure confirmation of isolated compounds

**FIGURE 1:**
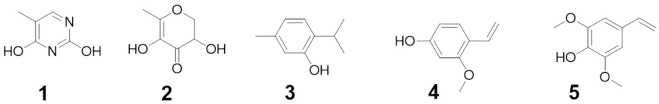
Structure of isolated compounds (compounds **1–5**).

The compounds obtained, purified, and identified from the chloroform fraction of *H. digitata* are shown in Figure 1. The compounds were characterized by GC-MS and NMR analyses. The phenolic compound **1** is chemically 5-methylpyrimidine-24,4-diol with the molecular formula C_5_H_6_N_2_O_2_ and a molecular weight of 126. The MS spectrum pattern of compound **1** was 126 (100%), 109 (17%), 95 (24%), 87 (19%), 82 (18%), 73 (43%), 70 (18%), 60 (80%), 55 (80%), and 43 (96%). The phenolic compound **2** is chemically 3,5-dihydroxy-6-methyl-2,3-dihydro-4H-pyran-4-one with the molecular formula C_6_H_8_O_4_ and a molecular weight of 144. The MS spectrum pattern of compound **2** was 144 (60%), 115 (04%), 101 (58%), 72 (38%), 55 (37%), and 43 (100%). The phenolic compound **3** is chemically 2-isopropyl-5-methylphenol with the molecular formula C_10_H_14_O and a molecular weight of 150. The MS spectrum pattern of compound **3** was 150 (15%), 135 (100%), 115 (19%), 107 (10%), 91 (22%), 77 (11%), 65 (04%), and 56 (04%). The phenolic compound **4** is chemically 3-methoxy-4-vinylphenol with the molecular formula C_9_H_10_O_2_ and a molecular weight of 150. The MS spectrum pattern of compound **4** was 150 (100%), 135 (83%), 121 (03%), 107 (77%), 89 (05%), 81 (19%), 77 (59%), 63 (07%), 51 (19%), and 43 (05%). The phenolic compound **5** is chemically 2,6-dimethoxy-4-vinylphenol with the molecular formula C_10_H_12_O_3_ and a molecular weight of 180. The MS spectrum pattern of compound **5** was 180 (100%), 175 (04%), 165 (40%), 137 (41%), 122 (22%), 109 (05%), 91 (08%), 77 (23%), 65 (20%), and 57 (16%).

### 3.2 *In vitro* antioxidant assay

#### 3.2.1 ABTS assay

The ABTS inhibitory activity of compounds 1 and 2 revealed dose-dependent activity (Table 1). In this assay, compound **1** demonstrated 91.58 ± 1.12, 87.65 ± 1.34, 84.90 ± 0.96, 79.03 ± 0.48, and 75.90% ± 0.48% inhibitions at concentrations of 1,000, 500, 250, 125, and 62.5 μg/mL, respectively. The IC_50_ value obtained from the dose–response curve calculations was 0.98 μg/mL. Compound **2** also displayed excellent potential, i.e., it revealed 86.47 ± 0.22, 81.94 ± 0.45, 77.61 ± 1.70, 72.64 ± 0.16, and 68.52% ± 0.38% inhibitions at 1,000–62.5 μg/mL concentrations with an IC_50_ value of 3 μg/mL, respectively. All the other compounds also exhibited well-to-moderate percent inhibition against ABTS assay (Table 1).

**TABLE 1:** ABTS, DPPH, and H_2_O_2_ assays of the compounds isolated from *H. digitata*.

Compound	Concentration (µg/mL)	ABTS % inhibition	IC_50_ (µg/mL)	H_2_O_2_% inhibition	IC_50_ (µg/mL)	DPPH % inhibition	IC_50_ (µg/mL)
Comp 1	1,000	91.58 ± 1.12*	0.98	85.20 ± 0.23^ns^	05	93.33 ± 0.49*	0.90
	500	87.65 ± 1.34***		81.13 ± 0.20^ns^		87.03 ± 0.23***	
	250	84.90 ± 0.96*		76.87 ± 1.27 ^ns^		83.00 ± 0.58**	
	125	79.03 ± 0.48**		71.76 ± 0.61 ^ns^		78.67 ± 0.89***	
	62.5	75.90 ± 0.48***		69.91 ± 1.30 ^ns^		75.00 ± 1.15***	
Comp 2	1,000	86.47 ± 0.22***	03	86.39 ± 0.60 ^ns^	06	91.62 ± 0.74***	04
	500	81.94 ± 0.45***		80.39 ± 0.49 ^ns^		86.86 ± 0.60***	
	250	77.61 ± 1.70***		75.36 ± 0.49 ^ns^		81.48 ± 0.64***	
	125	72.64 ± 0.16***		71.34 ± 0.55 ^ns^		76.54 ± 0.50***	
	62.5	68.52 ± 0.38***		67.90 ± 1.16 ^ns^		72.74 ± 0.61***	
Comp 3	1,000	87.79 ± 0.63***	21	77.34 ± 0.98***	33	81.03 ± 0.35***	17
	500	83.67 ± 0.61***		72.32 ± 1.06***		77.08 ± 0.47***	
	250	76.69 ± 0.77***		67.05 ± 0.75***		72.91 ± 0.88***	
	125	72.54 ± 0.50***		62.70 ± 1.25***		67.90 ± 0.96***	
	62.5	65.00 ± 0.30***		58.74 ± 0.68***		62.98 ± 0.72***	
Comp 4	1,000	75.33 ± 0.49***	42	69.58 ± 1.12***	65	77.73 ± 0.03***	40
	500	72.03 ± 0.23***		65.65 ± 1.34***		73.42 ± 0.12***	
	250	65.00 ± 0.58***		62.90 ± 0.96***		68.39 ± 0.35***	
	125	61.67 ± 0.89***		57.03 ± 0.48***		63.36 ± 0.71***	
	62.5	57.00 ± 1.15***		53.90 ± 0.48***		58.15 ± 0.22***	
Comp 5	1,000	79.49 ± 0.60***	30	76.42 ± 0.46***	35	75.28 ± 0.42***	16
	500	74.31 ± 0.58***		70.53 ± 0.41***		71.75 ± 0.21***	
	250	68.76 ± 0.61***		66.68 ± 0.64***		67.05 ± 0.13***	
	125	63.08 ± 1.04***		62.46 ± 0.47***		63.99 ± 0.19***	
	62.5	60.59 ± 0.30***		57.51 ± 0.62***		60.48 ± 0.23***	
AA	1,000	95.85 ± 0.18	0.12	87.50 ± 2.26	02	97.53 ± 0.20	0.47
	500	91.59 ± 0.30		83.01 ± 0.42		93.62 ± 0.17	
	250	87.75 ± 0.14		78.07 ± 0.62		88.42 ± 0.11	
	125	84.47 ± 0.49		73.70 ± 0.35		84.20 ± 0.15	
	62.5	81.12 ± 0.34		71.73 ± 0.66		81.35 ± 0.18	

Data are represented as mean ± SEM; IC_50_ calculated for positive control (ascorbic acid). The data are presented as the mean ± standard error mean. A two-way ANOVA was conducted, followed by the Bonferroni test. Values that showed significant differences compared to the positive control are indicated as follows: *n* = 3, * = *p* < 0.05, ** = *p* < 0.01, and *** = *p* < 0.001; ns indicates non-significant results.

#### 3.2.2 DPPH and H_2_O_2_ assay

The DPPH and H_2_O_2_ inhibitory activities of compounds isolated from *H. digitata* illustrated dose-dependent antioxidant activity, as shown in Table 1. Compound **1** again revealed prominent activity with 93.33 ± 0.49, 87.03 ± 0.23, 83.00 ± 0.58, 78.67 ± 0.89, and 75.00% ± 1.15% inhibitions against DPPH, while 85.20 ± 0.23, 81.13 ± 0.20, 76.87 ± 1.27, 71.76 ± 0.61, and 69.91% ± 1.30% against H_2_O_2_ at concentrations of 1,000, 500, 250, 125, and 62.5 μg/mL, respectively. The IC_50_ values calculated were 0.9 and 5 μg/mL for compound 1 against DPPH and H_2_O_2_, respectively. The IC_50_ value for ascorbic acid was noted as 0.47 and 2 μg/mL (Table 1). All the other three compounds also displayed excellent result against free radicals.

#### 3.2.3 *In vitro* anti-inflammatory assay

##### 3.2.3.1 COX-2 assay

The activity of isolated compounds, celecoxib and indomethacin, inhibiting COX-2 enzymes was evaluated using IC_50_ values (µg/mL), which are defined as the concentration that causes 50% enzyme inhibition. The results of our investigations are presented in Table 2. All the test compounds inhibited the COX-2 enzymes, but compounds **1** and **2** showed a significant percent inhibition. The IC_50_ values (10.70 μg/mL and 14.51 μg/mL, respectively) of compound **1** and compound **2** revealed that both the compounds have a significant inhibitory effect of COX-2 enzymes, as compared to other compounds. The IC_50_ value of celecoxib (3.22 μg/mL), being a standard drug, showed a more potent inhibitory effect on COX-2 enzymes. The IC_50_ values of compounds **3, 4**, and **5** were 21.72, 39.06, and 20.29 μg/mL, respectively. Other compounds showed a weak COX-2 inhibitory effect. Thus, our research findings showed that compounds **1** and **2** have a potent COX-2 inhibitory effect.

**TABLE 2:** Percentage of COX-2 and 5-LOX inhibition activity of isolated compounds.

S. No	Concentration (μg/mL)	% COX-2 inhibition	IC_50_ μg/mL	% COX-1 inhibition	IC_50_ μg/mL	% 5-LOX inhibitions	IC_50_ μg/mL
Comp 1	1,000	83.13 ± 0.80***	10.70	73.08 ± 1.04***	42.76	87.63 ± 0.64**	7.40
	500	78.83 ± 0.73***		66.45 ± 0.90***		82.45 ± 0.55***	
	250	72.70 ± 0.51***		60.58 ± 0.63***		76.53 ± 0.41***	
	125	66.43 ± 0.70***		55.40 ± 0.20***		71.42 ± 0.46***	
	62.50	61.06 ± 0.70***		45.80 ± 0.90***		65.68 ± 0.64***	
Comp 2	1,000	80.85 ± 0.18***	14.51	71.37 ± 0.56***	110	83.53 ± 0.20***	
	500	75.59 ± 0.30***		65.29 ± 0.41***		78.62 ± 0.17***	
	250	68.75 ± 0.14^***^		54.58 ± 0.58^***^		73.42 ± 0.11^***^	
	125	63.47 ± 0.49***		46.39 ± 0.62***		66.20 ± 0.15***	
	62.50	58.12 ± 0.34***		40.83 ± 1.06***		61.35 ± 0.18***	
Comp 3	1,000	79.00 ± 0.16***	21.72	65.17 ± 0,72***	185.83	73.39 ± 0.60***	
	500	74.66 ± 1.20***		57.85 ± 0.97***		67.39 ± 0.49***	
	250	66.33 ± 0.33***		51.37 ± 1,65***		61.36 ± 0.49***	
	125	62.50 ± 0.44***		46.73 ± 0.78***		57.34 ± 0.55***	
	62.50	53.00 ± 0.57***		41.34 ± 1.01***		51.90 ± 1.16***	
Comp 4	1,000	85.72 ± 0.79***	20.29	71.33 ± 0.49***	218.83	84.83 ± 0.62***	27.35
	500	77.68 ± 0.63***		63.03 ± 0.23***		80.76 ± 0.63***	
	250	71.46 ± 0.53***		49.00 ± 0.58***		75.70 ± 0.62***	
	125	64.78 ± 0.60***		42.67 ± 0.89***		66.65 ± 0.78***	
	62.50	55.56 ± 0.52***		33.00 ± 1.15***		59.81 ± 0.65***	
Comp 5	1,000	74. 4 ± 0.68***	39.06	67.73 ± 0.03***	277.91	76.7 ± 0.66***	30.75
	500	66.2 ± 0.73***		57.42 ± 0.12***		71.3 ± 1.11***	
	250	61.0 ± 0.33***		47.39 ± 0.35***		65.5 ± 1.04***	
	125	56.4 ± 0.63***		41.36 ± 0.71***		57.2 ± 0.57***	
	62.50	46.9 ± 0.42***		29.15 ± 0.22***		49.9 ± 0.65***	
Montelukast	1,000	—	—	—	—	93.55 ± 0.40	4.50
	500					89.37 ± 1.65	
	250					85.50 ± 0.40	
	125					79.60 ± 0.90	
	62.50					74.17 ± 0.72	
Celecoxib	1,000	95.20 ± 0.15	3.22	—	—	—	—
	500	91.17 ± 0.53					
	250	86.98 ± 0.85					
	125	81.20 ± 0.65					
	62.50	77.80 ± 0.37					
Indomethacin	1,000	—	—	78.39 ± 0.49	17.69	—	—
	500			73.47 ± 0.52			
	250			67.44 ± 0.55			
	125			61.40 ± 0.51			
	62.50			55.57 ± 0.84			

The data are presented as the mean ± standard error mean. A two-way ANOVA was conducted, followed by the Bonferroni test. Values that showed significant differences compared to the positive control are indicated as follows: n = 3, * = *p* < 0.05, ** = *p* < 0.01, and *** = *p* < 0.001; ns indicates non-significant results.

##### 3.2.3.2 COX-1 assay

COX-1 enzymes assays’ results showed that all the investigated compounds showed a moderate-to-weak inhibitory effect of COX-1 enzymes. The research findings showed IC_50_ values of compounds **1** and **2** (42.76 and 110 μg/mL, respectively), whereas compound **3, 4,** and **5** have IC_50_ values of 185.83, 277.91, and 218.83 μg/mL, respectively. However, the IC_50_ value of indomethacin was found to be 17.69 μg/mL. Our result findings showed that all the isolated compounds exhibited a moderate COX-1 enzyme inhibitory effect (Table 2).

##### 3.2.3.3 5-LOX assay

*In vitro* lipoxygenase (5-LOX) inhibitory assays’ data revealed that two compounds (**1** and **2**) have stronger 5-LOX inhibitory action. Different concentrations of the isolated compounds showed the percent inhibition. The highest percent inhibition of LOX-5 enzymes assays was reported for compound **1** (87.63 ± 0.64 at 1,000 μg/mL concentration with IC_50_: 7.40 μg/mL) and compound **2** (83.53 ± 0.20 at 1,000 μg/mL concentration with IC_50_: 10.65 μg/mL). The highest percent inhibition for montelukast was found to be 93.55 ± 0.40 at 1,000 μg/mL concentration with IC_50_: 4.5 μg/mL. However, compounds **3, 4,** and **5** have moderate LOX-5 inhibitory action with a minimal percent inhibition of 73.39 ± 0.60 at 1,000 μg/mL concentration with IC_50_ 26.18 μg/mL, 76.7 ± 0.66 at 1,000 μg/mL concentration with IC_50_ 30.75 μg/mL, and 84.83 ± 0.62 at 1,000 μg/mL concentration with IC_50_ 27.35 μg/mL (Table 2).

#### 3.2.4 *In vivo* antioxidant assays

##### 3.2.4.1 Effects of phenolic compound 1 on the SOD activity of mice

**FIGURE 2:**
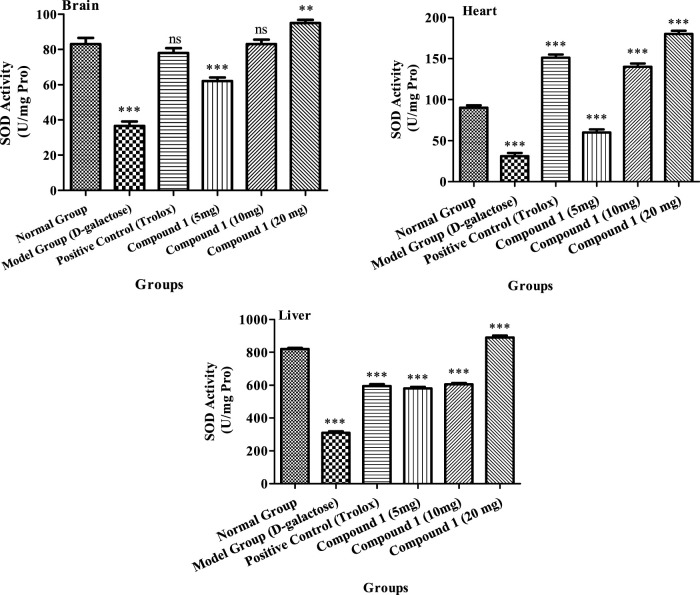
SOD activity of the brain, heart, and liver of mice in different treatment groups. Different lowercase letters mean significant difference in SOD activity between the same organ among treatment groups, and two-way ANOVA was conducted, followed by the Bonferroni test. Values that showed significant differences compared to the positive control are indicated as follows: n = 3, * = *p* < 0.05, ** = *p* < 0.01, and *** = *p* < 0.001; ns indicates non-significant results.

The superoxide dismutase (SOD) activity in experimental mice measured at 36.5 U/mL, 31.0 U/mg protein, and 310 U/mg protein in the brain, heart, and liver, respectively, which were considerably less, as compared to the control group (*p* < 0.05, Figure 2), suggests the establishment of the successful aging model. In contrast, with the comparison with the model group, the activity of SOD in the Trolox and tested compound groups was higher compared to the model group (*p <* 0.05), suggesting that Trolox and compound **1** had antioxidant capacity (*p* < 0.05). In the compound **1** (10 mg) group, SOD activities in the heart were 140.0 U/mg proteins that were not considerably different (*p* > 0.05) from the positive control group. The activities of SOD in the high-dosage group’s brain, heart, and liver, on the other hand, were 95 U/mL, 180.0 U/mg proteins, and 890.0 U/mg protein, respectively, substantially greater than those in the remaining groups (*p* < 0.05). Our findings revealed that compound **1** might reduce oxidative stress in the body caused by D-galactose while also increasing the SOD activity.

##### 3.2.4.2 Estimation of GSH-Px

**FIGURE 3:**
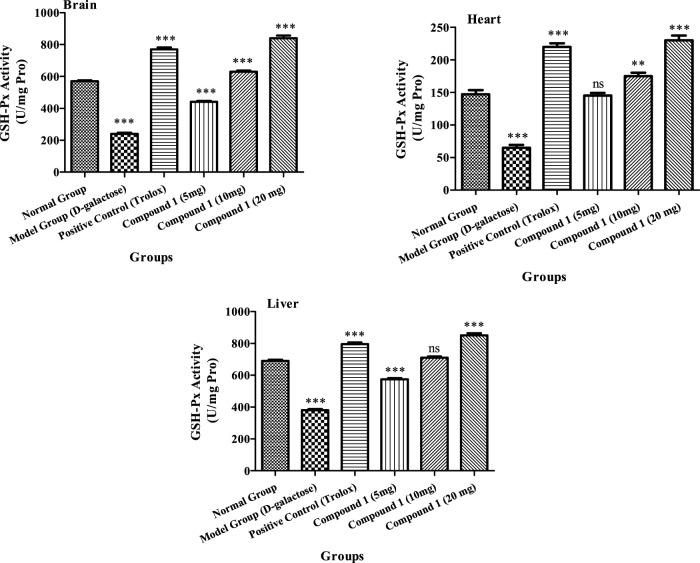
GSH-Px activity in the brain, heart, and liver of mice among treatment groups. Different lowercase letters mean significant difference in GSH-Px activity between the same organ of different treatment groups (*p* < 0.05), and two-way ANOVA was conducted, followed by the Bonferroni test. Values that showed significant differences compared to the positive control are indicated as follows: n = 3, * = *p* < 0.05, ** = *p* < 0.01, and *** = *p* < 0.001; ns indicates non-significant results.

The activity GSH-PX was higher in the brain, heart, and liver in the higher-dose groups of the investigated compound **1** than in the other groups’ brain, heart, and liver (*p* < 0.05, Figure 3). The GSH-Px activity in heart and liver tissues of animals increased significantly in the positive control group, in comparison to the control and aging model groups (*p* < 0.05). The brain GSH-Px activity of low-dosage and control groups was not significant (*p* > 0.05). Furthermore, activity of GSH-Px in the brain, heart, and liver was 240, 65, and 380 U/mg protein, respectively, indicating that the aging model groups showed reduced GSH-Px, as compared to other groups (*p* < 0.05). As a result, compound **1** enhances the activity of GSH-Px, and the antioxidant impact is associated with strain concentration Figure 3.

##### 3.2.4.3 Estimation of the MDA level

**FIGURE 4:**
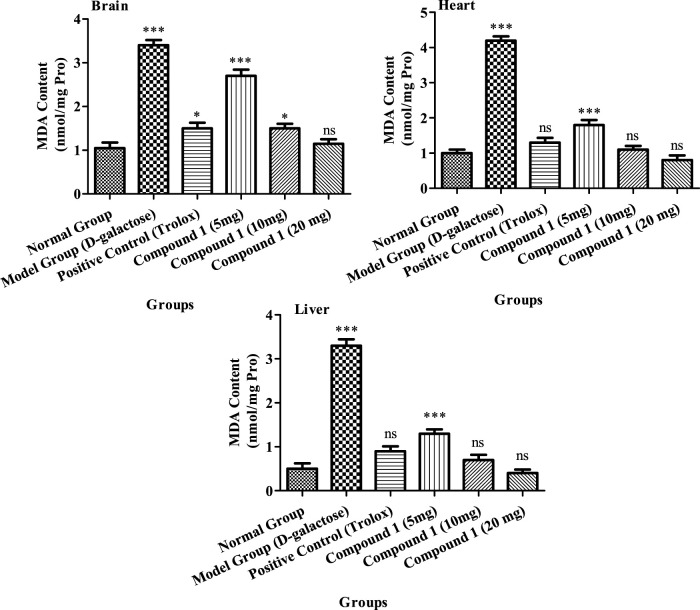
MDA activity in the brain, heart, and liver of mice among treatment groups. Different lowercase letters mean a significant difference in MDA activity between the same organ of different treatment groups, and two-way ANOVA was conducted, followed by the Bonferroni test. Values that showed significant differences compared to the positive control are indicated as follows: n = 3, * = *p* < 0.05, ** = *p* < 0.01, and *** = *p* < 0.001; ns indicates non-significant results.

MDA levels were considerably greater in aging model group’s serum, heart, and liver than the control group (*p* < 0.05, Figure 4). The MDA concentration of animals treated with compound 1 (20 mg) was 1.15 nmol/mL in the brain, and 0.8 and 0.4 nmol/mg protein in the heart and liver, respectively, which was notably less than other groups (*p* < 0.05). The concentration of MDA in the positive control and compound **1** (10 mg) groups did not differ significantly (*p* > 0.05).

##### 3.2.4.4 Estimation of CAT activity

**FIGURE 5:**
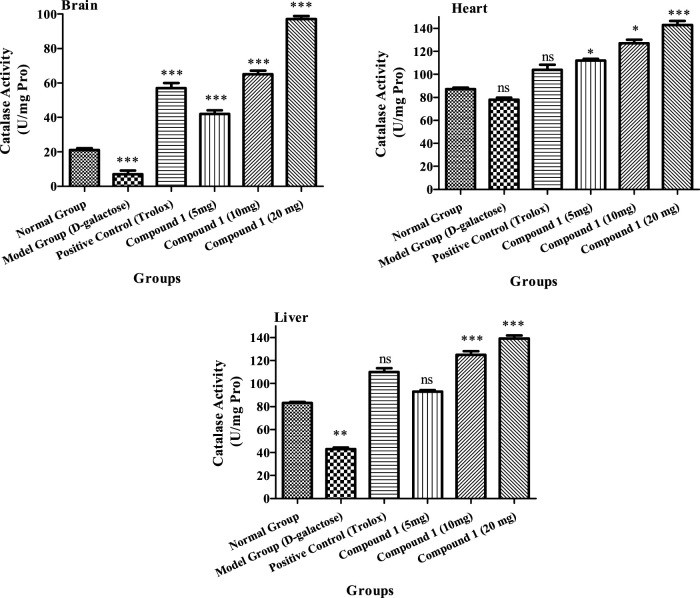
CAT activity of the brain, heart, and liver of mice in different treatment groups. Different lowercase letters mean a significant difference in CAT activity between the same organ among treatment groups, and two-way ANOVA was conducted, followed by the Bonferroni test. Values that showed significant differences compared to the positive control are indicated as follows: n = 3, * = *p* < 0.05, ** = *p* < 0.01, and *** = *p* < 0.001; ns indicates non-significant results.

The CAT activities of the brain, heart, and liver of the high tested dose were 97 U/mL, 143.02 U/mg protein, and 139.0 U/mg protein, respectively, as shown in Figure 5, which were higher than the CAT activities (*p* < 0.05) of the other treated groups, demonstrating that the strain concentration produces a significant impact on CAT activities. The aging model group, on the other hand, had decreased CAT activity in the brain, heart, and liver of mice in comparison to other groups (*p* < 0.05). In the brain, heart, and liver of mice, the CAT activity of positive control was more than the control and aging model groups (*p* < 0.05). Our findings show that compound **1** might increase CAT activity in the brain, heart, and liver tissues of mice.

#### 3.2.5 *In vivo* anti-inflammatory activity

##### 3.2.5.1 Carrageenan-induced inflammation

In carrageenan-induced inflammation, the anti-inflammatory action of the complete chemically produced molecule was remarkable. The anti-inflammatory efficacy of all substances was dose-dependent and considerable. Compound **1** demonstrated the greatest anti-inflammatory effect at 5, 10, and 20 mg/kg doses. Compound **1** has outstanding anti-inflammatory activity (54.27% ± 3.36%) at a maximum dose of 20 mg/kg, which peaked at the 5th hour after carrageenan insertion and remained significant (***p < 0.001) throughout the investigated drug administration (Table 3). Administration of aspirin (5 mg/kg) for 5 h had a significant effect (58.62 1.53%, ***p < 0.001), which was roughly half of the effects caused by compound 1 at 20 mg/kg.

**TABLE 3:** Percent inhibition of compounds in the carrageenan-induced paw edema.

Treatment	Dose (mg/kg)	Percentage inhibition of paw edema				
		1 h	2 h	3 h	4 h	5 h
Vehicle	**—**	7.642 ± 3.73	7.372 ± 1.46	11.43 ± 1.07	14.47 ± 1.59	7.317 ± 3.22
Aspirin	5	47.50 ± 1.10***	53.61 ± 1.45***	54.24 ± 1.34***	57.90 ± 1.93***	58.62 ± 1.53***
Compound 1	5	46.72 ± 2.63 ns	50.46 ± 2.52**	53.58 ± 1.33**	52.37 ± 1.46**	55.23 ± 1.61***
	10	51.37 ± 2.23***	53.56 ± 3.57***	56.56 ± 1.57***	55.76 ± 2.62***	61.68 ± 2.77***
	20	54.27 ± 3.06***	56.38 ± 1.67***	59.74 ± 2.67***	58.02 ± 1.95***	63.57 ± 1.46***

Percentage inhibition caused by compound 1 (25, 50, and 100 mg/kg) using a mouse model of carrageenan-induced paw edema. Each percentage point, represented as mean ± SEM, was calculated for each group of eight mice. Statistical analysis involved two-way ANOVA, followed by Bonferroni’s *post hoc* test. Asterisks show significant differences from the control (vehicle) group, with *p < 0.05, **p < 0.01, and ***p < 0.001, while “ns" denoted non-significant results. Each group consisted of n = 8 mice.

##### 3.2.5.2 Anti-inflammatory mechanism of compound 1

**FIGURE 6:**
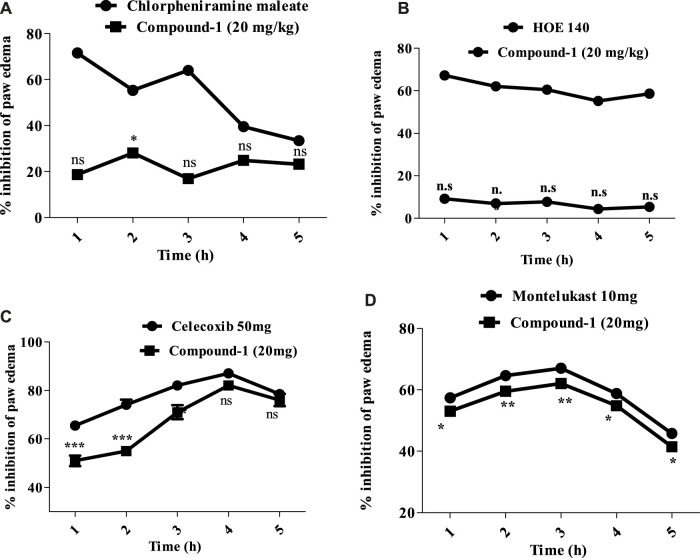
**(A)** Percentage inhibition produced by histamine and compound 1 (20 mg/kg) in the histamine-induced paw edema model. **(B)** Percentage inhibition produced by bradykinin and compound 1 (20 mg/kg) in bradykinin-induced paw edema. **(C)** Percentage inhibition produced by tested compound 1 (20 mg/kg) in prostaglandin E2-induced paw edema. **(D)** Percentage inhibition produced by tested compound 1 (20 mg/kg) in leukotriene-induced paw edema. Each percentage point signifies mean ± SEM for a group of eight mice. Statistical analysis involved ANOVA, followed by Dunnett’s *post hoc* test. Asterisks denote significance: *p < 0.05, **p < 0.01, and ***p < 0.001; “ns” indicates non-significant values.

Compound **1** was the most active compound among the other compounds both *in vitro* and *in vivo*. To determine the mechanism involved, different inflammatory agents were employed, i.e., histamine, bradykinin, prostaglandin, leukotriene, and bradykinin. Compound **1** had a mild anti-histaminic effect (28.12 ± 0.62) at a high dose (20 mg/kg) at the 2^nd^ hour, which may be because of compound **1** inhibiting the mast cells to release mediators. Moreover, the reference drug (chlorpheniramine maleate) exhibited significant inhibition of the edema induced by histamine at 1 h, resulting in a reduction to 71.62 ± 1.16 (Figure 6A).

##### 3.2.5.3 Effect of compound 1 on bradykinin-induced inflammation

Following the administration of bradykinin injection, edema developed in the mice paw, peaking 60 min post-injection. Compound 1 exhibited no activity against paw edema at low doses. At the dose of 20 mg/kg, compound 1 reduced paw edema 9.20 ± 1.12 during the initial hour, although not statistically significant in comparison with the standard drug (HOE 140) Figure 6B.

##### 3.2.5.4 Effect of compound 1 on prostaglandin E_2_ (PGE_2_) inflammation

The level of prostaglandin E_2_ significantly increased in the paw tissue after injecting the prostaglandin E_2_ mediator, as shown in Figure 6C. The treatment of mice with compound **1** (20 mg/kg) while celecoxib (50 mg/kg) caused a significant reduction of the increased PGE _2_ level. The effect of compound **1** reached a maximal level at the 4^th^ hour (82.0 ± 0.34), compared with celecoxib, and remained significant still the 5th hour (***p ˂ 0.001).

##### 3.2.5.5 Effect of compound 1 on leukotriene inflammation

Leukotriene administration triggered inflammation and induced edema in mouse paws, with swelling reaching its peak 30 min after leukotriene exposure. Compound 1 demonstrated significant activity against mouse paw edema. Compound **1** at a dose of 20 mg/kg inhibited swelling of paw 53.00 ± 1.10, 59.50 ± 0.92, 62.0 ± 0.70, 54.84 ± 0.92, and 41.43 ± 1.10 at 1, 2, 3, 4, and 5^th^ hours, respectively. The effect of compound **1** at the 1st hour was significant (**p ˂ 0.01) and achieved a maximum effect at a 3rd hour after exposure to the leukotriene mediator and remain significant (***p ˂ 0.001) up to 5th hour. Montelukast demonstrated a % inhibition of 67.02 ± 1.74 at the 3rd hour (Figure 6D).

#### 3.2.6 *In silico* studies

Docking studies were carried out to determine the pharmacological characteristics of the isolated compounds upon binding with specific protein targets, focusing on the enzyme–ligand interaction through the induced fit model. These findings were examined to know the diverse interaction parameters. The binding interactions of all isolated compounds, in addition to reference drugs, are expressed in Table 4.

**TABLE 4:** Binding affinity of isolated compounds and standard drugs at specific target sites.

Compound	COX		5-LOX
	COX-I	COX-II	3V92
	4O1Z	5F1A	
1	−6.7	−6.5	−6.7
2	−6.1	−5.5	−5.9
3	−6.3	−6.2	−5.9
4	−6.4	−6.1	−5.7
5	−5.9	−6.1	−6.1
Montelukast	—	—	−8.7
Celecoxib	—	−8.1	—
Indomethacin	−7.5	—	—

**FIGURE 7:**
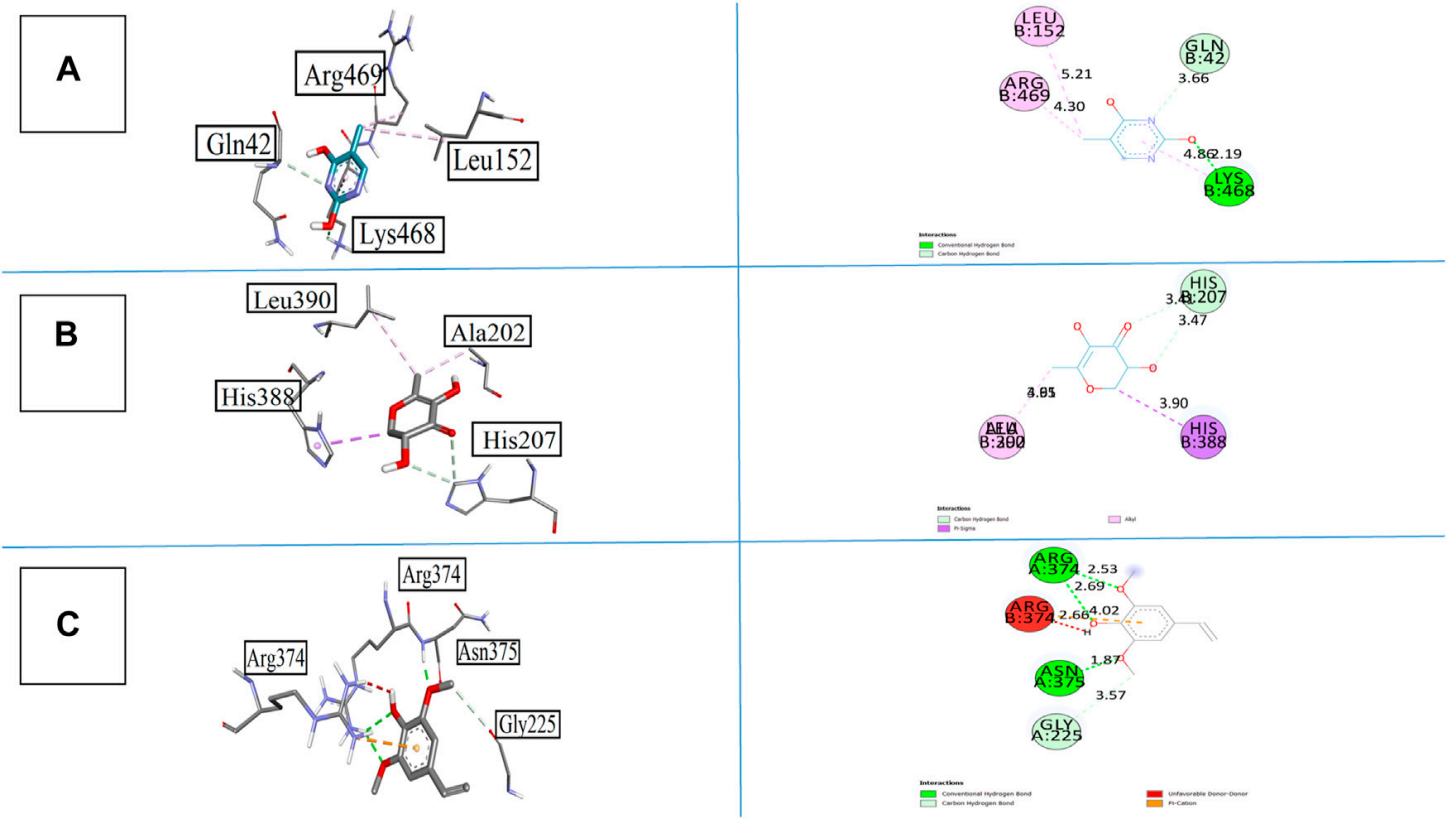
3D and 2D visualization of synthesized compounds with cyclooxygenase COX-I, **(A)** compound 1, **(B)** compound 2, and **(C)** compound 5.

Compound **1** emerged as the most active compound, displaying −6.7 kcal/mol binding energy against COX-I and −6.5 kcal/mol against COX-II. The interactions of all synthesized compounds are analyzed against cyclooxygenase-I, and results are illustrated in Figures 7A–C. Compound **1** gave the strong interactions with amino acid residues as Gln42, Leu152, Lys468, and Arg469 inside the active site, with one conventional hydrogen bond, one carbon hydrogen bond, and two alkyl–pi–alkyl bonds at the bond lengths of 2.19, 3.66, 5.21, and 4.30 Å, respectively. Similarly, when compound **2** was analyzed against cyclooxygenase-I, it provided a carbon–hydrogen bond via a keto and hydroxyl group with His207 at the bond length of 3.41 and 3.47 Å. It also displayed a pi–sigma and alkyl bond with His388 and Ala202 through the heterocyclic ring at the bond length of 3.90 and 4.05 Å, respectively. When compound **5** was visualized inside the same active site, it displayed two conventional hydrogen bonds with Arg374 and Asn375, one carbon hydrogen bond with Gly225 and one prominent pi–cation interaction with Arg374 that predominated the pharmacological role of this compound.

**FIGURE 8:**
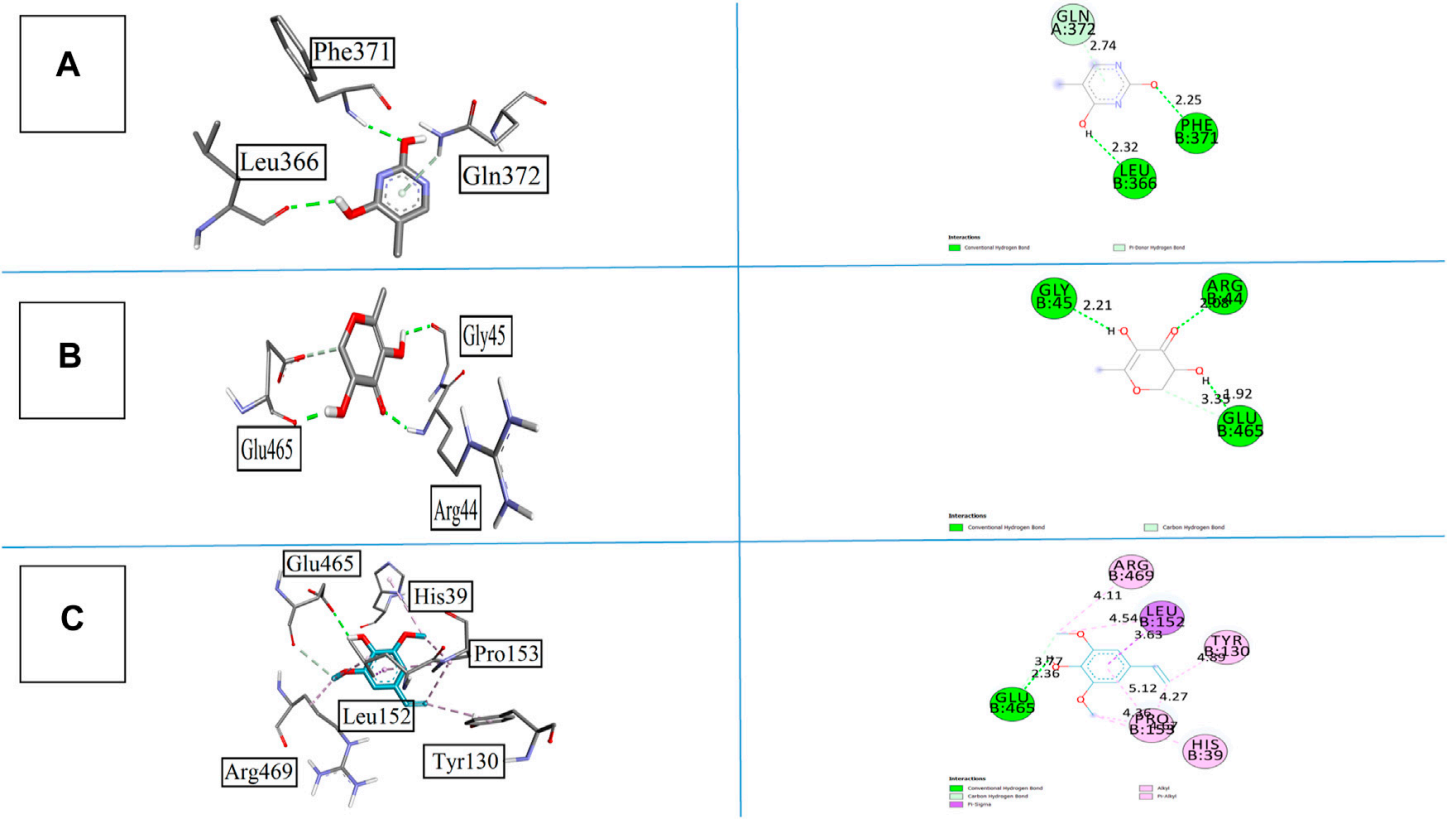
3D and 2D visualization of isolated compounds with cyclooxygenase COX-II, **(A)** compound 1, **(B)**compound 2, and **(C)** compound 5.

The binding affinities of all synthesized compounds were analyzed against cyclooxygenase-II with pdb 5F1A. All the compounds gave satisfactory results, but compound **1** was more prominent in this regard. It gave two conventional hydrogen bonds with Phe371 and Leu366 at the bond lengths of 2.25 and 2.32 Å, respectively, along with one pi-donor hydrogen bond that exaggerates the results. Compound **2** having the heterocyclic ring with keto and hydroxyl groups displayed three conventional hydrogen bonds with Arg44, Gly45, and Glu465 at the bond lengths of 2.08, 2.21, and 3.35 Å, respectively. The bonding affinity was found to be −5.5 kcal/mol. All the interactions are displayed in Figures 8A–C. Compound **5** also provided noticeable results with a binding energy of −6.1 kcal/mol with COX-II. The interactions were more interesting in this case as it showed one conversional hydrogen bond between the hydroxyl group and amino acid Alu465 at the bond length of 2.36 Å. Furthermore, it displayed pi–sigma and pi–alkyl interactions with Leu152, His39, Try130, pro153, and Arg469 at the bond lengths of 3.63, 4.36, 4.89, 4.11, and 4.27 Å, respectively. The standard drug celecoxib displayed the binding affinity of −8.1 kcal/mol.

**FIGURE 9:**
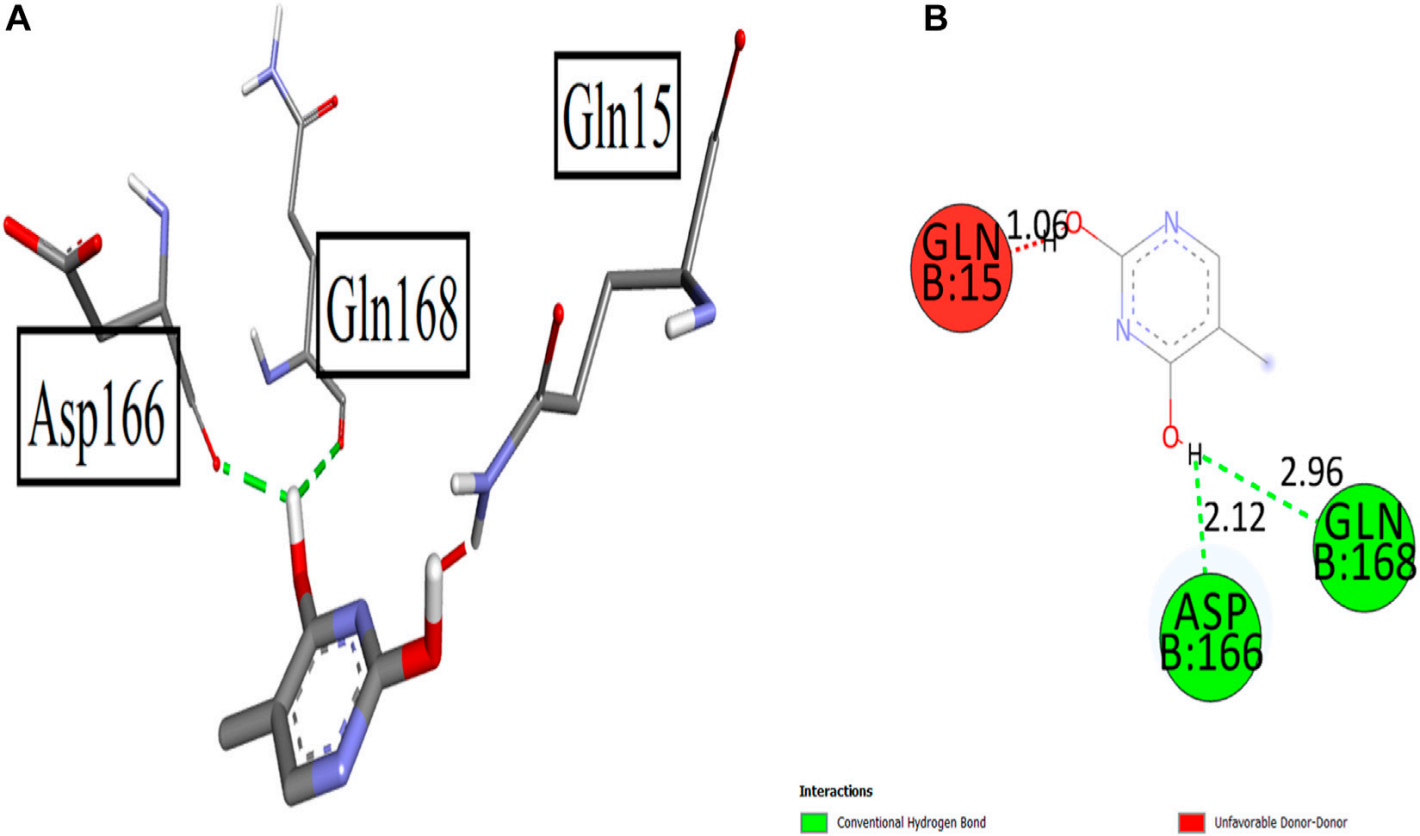
3D **(A)** and 2D **(B)** interactions of the synthesized compound 1 with 5-lipoxygenase 5-LOX.

All synthesized compounds including the standard control drugs were allowed to dock with the 5-lipoxygenase enzyme having pdb 3V92. Results were satisfying as all the synthesized compounds gave binding affinities with good values. Compound **1** displayed excellent results with a binding score of −6.7 kcal/mol with this receptor. Relatively, the structural alterations and functional groups of compound 1 allowed it to give more binding affinity toward the receptor. It yielded two strong conventional hydrogen bonds with Asp166 and Gln168 with bond lengths of 2.12 and 2.96 Å, respectively. These connections were displayed with the hydroxyl group attached to the ring system. Figure 9 elaborates the interactions in this case.

**FIGURE 10:**
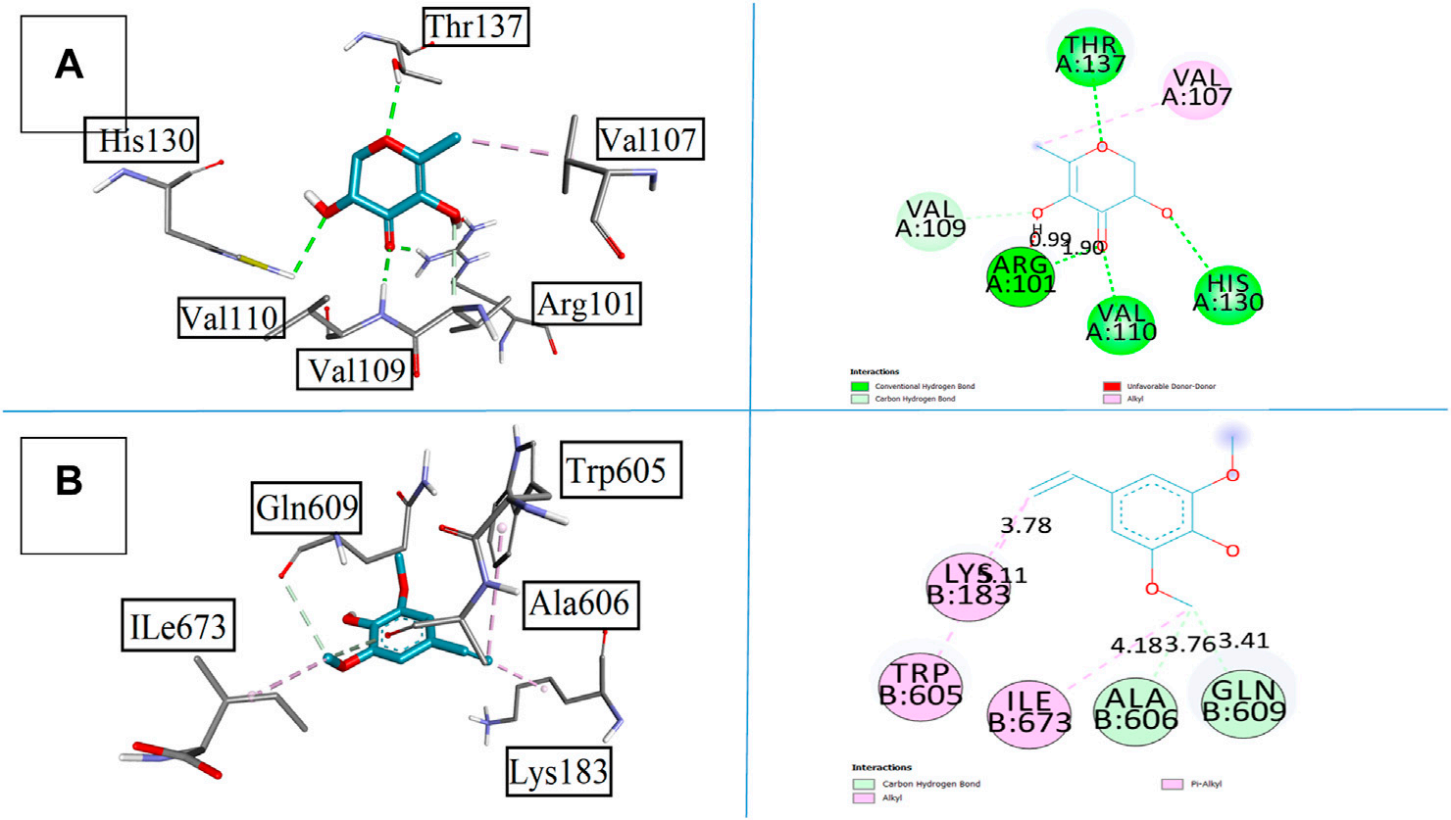
Presenting the 3D and 2D visualization of synthesized compounds with 5-lipoxygenase 5-LOX, **(A)** compound 2, and **(B)** compound 5.

In relation to these pharmacological behaviors, compounds **2** and **5** were the strong candidates that yielded results close to the standard drug montelukast. Compound **2** showed the four conventional bonds with amino acid residues as Arg101, Val110, His130, and Thr137 with bond lengths of 0.99, 1.90, 1.39, and 1.49 Å, respectively. The binding affinity was found to be −5.9 kcal/mol. Other prominent residues were observed as Val109 with the carbon hydrogen bond and Val107 with the pi–alkyl bond. Compound **5** has the methoxy group along with hydroxyl functional groups and unsaturation that increase the binding behavior with the targeted receptor. It formed alkyl and pi–alkyl interactions with Lys183, Trp605, and Ile673 with the bond lengths of 3.78, 3.11, and 4.18 Å, respectively. Other prominent amino acid residues involved in affinities were Ala606 and Gln609 with bond lengths of 3.76 and 3.41 Å accordingly that make this compound more feasible to be used as a good pharmacological agent. All results are elaborated in Figure 10.

## 4 Discussion

The D-galactose induction model of subacute aging mice is well-known and extensively utilized (Kong et al., 2018). It comprises a continual administration of D-galactose, converted inside the cells by galactose reductase to galactose, leading in a shift in osmotic pressure, followed by enlargement of cells and aging (Kumar et al., 2022). Whenever the body’s cells are exposed to acute oxidative stress, cytoplasmic enzymes which reduce oxidative enzymes, i.e., CAT, SOD, and GSH-Px, are insufficient to remove ROS (Angwa et al., 2022). It is a simple and efficient way to investigate antioxidant potential in the D-galactose-induced subacute aging model and compare antioxidant potential of enzymes to the control group (Gong et al., 2023). CAT, SOD, and GSH-Px are examples of body’s free radical scavenging enzyme defense systems that work together to scavenge hydrogen peroxide, superoxide radicals, and hydroxyl radicals, respectively (Hassan et al., 2017). SOD may convert superoxide radicals to H_2_O_2_, which is still harmful, and use the Fenton reaction to generate hydroxyl radicals. The hydroxyl radical is capable of rapidly reacting with organic molecules in the cell and having a damaging effect. Furthermore, CAT breaks down hydroxyl free radicals to aid antioxidant defense in cells. Antioxidant enzymes, like GSH-Px, can be created intracellular under healthy circumstances to prevent them from oxidative stress (Bratovcic, 2020). Compound **1** dramatically increased the activities of SOD, GSH-Px, and CAT in the brain, heart, and liver of mice, respectively, in this investigation, demonstrating that compound **1** reduces the oxidative stress produced by D-galactose. It may also produce a synergistic effect with SOD, CAT, and GSH-Px and alleviate oxidative damage by scavenging free radicals. According to our study, compound **1** has an antioxidant potential in mice and is a potential plant-derived antioxidant. It is typically employed in the research of sausage starters or functional items as a bio-source antioxidant.

Chronic inflammation is a feature of several clinical disorders, including gastritis, atherosclerosis, rheumatoid arthritis, cancer, and inflammatory bowel disease. A wide variety of anti-inflammatory medications were used to cure these disorders, but they are highly toxic and have a variety of side effects. Hence, it is essential to explore non-toxic synthetic substances with anti-inflammatory properties (Mahmood et al., 2022).

The present study’s findings clearly show that isolated compounds have powerful anti-inflammatory potential both *in vitro* and *in vivo*, specifically *in vitro* LOX-5 and COX inhibitory assays, as well as *in vivo* carrageenan-induced inflammation in mice. In addition, numerous phlogistic agents (prostaglandin, leukotriene, histamine, and bradykinin) have been used to corroborate the mechanism of the effective compound.

COX-2 and 5-LOX are two major enzymes involved in the conversion of arachidonic acid to prostaglandin and leukotriene (Mahnashi et al., 2022b). Both of these enzymes are inhibited, resulting in a wide range of anti-inflammatory effects. For 5-LOX and COX-2 inhibition, the synthesized compounds were tested *in vitro*, utilizing a COX-2/1- and 5-LOX-catalyzed prostaglandin and leukotriene production assay. In comparison to other compounds investigated for COX-2/1 and 5-LOX inhibitory action *in vitro*, **compound 1** at dose of 1,000 g/mL was reported to have a prominent inhibitory response against COX-2 and 5-LOX. The carrageenan-induced paw edema is a biphasic, transitory, and time-dependent permeability and inflammatory reaction. Histamine is secreted in modest amounts by basophils during inflammatory processes. Just at the 2nd hour after histamine injection, compound **1** demonstrated a 28.101.64% anti-histaminic action, which is due to compound **1**’s mast cell-stabilizing effect. After activation, mediators are produced, increasing the vascular permeability and dilatation of vessels in the early stages of inflammation. Bradykinin enhanced microvascular permeability significantly. The inhibiting effects of bradykinin-induced inflammation may not be inhibited by compound **1**.

The major regulators of inflammation are prostaglandin E2 and leukotriene mediators. These two mediators were tested in the mouse paw. This option convinces us to seek out the most likely corridor of compound **1** as anti-inflammatory potency. We discovered that compound **1** greatly reduced the inflammatory prostaglandin E2 (PGE2)-induced paw edema in the existing investigation. Inflammation generated by leukotrienes yielded similar outcomes (Alqahtani et al., 2022). These data imply that compound **1** has dual inhibitory properties and has the ability to inhibit both LOX and COX pathways, which is supported by compound 1’s modest anti-histaminic action.

The molecular docking simulation is the tool used to find the binding interactions of the compounds within the protein site of the target enzyme (Ahmad et al., 2019; Javed et al., 2021; Sadiq et al., 2022). The docking simulations within the COX-2 isozyme’s binding region indicated that all the drugs had a hydrogen bond interaction with the selectivity pocket’s amino acid residues (Val523 and Ser353). The estimated binding energy values match the experimental results.

## 5 Conclusion

Herein, we have isolated five different phenolic compounds (**1–5**) from *H. digitata*. All the compounds, specifically compound 1, showed very practical *in vitro* antioxidant and anti-inflammatory potentials. Based on this high potency of compound **1**, it was subjected to extensive *in vivo* antioxidant and anti-inflammatory studies. The compound was also dominant in *in vivo* results. Furthermore, compound **1** also showed a very ideal response to different inflammatory and antioxidant markers. Our study suggests that the isolated compounds can treat inflammatory response by a multi-target approach.

## Data Availability

The original contributions presented in the study are included in the article/Supplementary Material; further inquiries can be directed to the corresponding authors.
